# Economics and Preventing Hospital-acquired Infection

**DOI:** 10.3201/eid1004.020754

**Published:** 2004-04

**Authors:** Nicholas Graves

**Affiliations:** * Centre for Health Research and Public Health, Queensland University of Technology, Kelvin Grove, Brisbane, Australia

**Keywords:** Cross infection, Infection Control, Economics, Opportunity Cost, Cost-Effectiveness Analysis

## Abstract

The economics of preventing hospital-acquired infections is most often described in general terms. The underlying concepts and mechanisms are rarely made explicit but should be understood for research and policy-making. We define the key economic concepts and specify an illustrative model that uses hypothetical data to identify how two related questions might be addressed: 1) how much should be invested for infection control, and 2) what are the most appropriate infection-control programs? We aim to make explicit the economics of preventing hospital-acquired infections.

Approximately 1 in 10 hospitalized patients will acquire an infection after admission, resulting in substantial economic cost (*1*). The primary cost is that patients with hospital-acquired infections have their stay prolonged, during which time they occupy scarce bed-days and require additional diagnostic and therapeutic interventions (*2*). Estimates of the cost of these infections, in 2002 prices, suggest that the annual economic burden is $6.7 billion per year in the United States (*3*)^1^ and £1.06 billion (approximately $1.7 billion) in the United Kingdom (*4*).

The economic rationale for preventing hospital-acquired infections has been discussed (*5*,*6*) and can be summarized as follows: hospital-acquired infections take up scarce health sector resources by prolonging patients’ hospital stay; effective infection-control strategies release these resources for alternative uses. If these resources have a value in an alternative use, then the infection control programs can be credited with generating cost savings; these infection control programs are costly themselves, so the expense of infection control should be compared to the savings.

For many hospital infections, the costs of prevention are likely to be lower than the value of the resources released (*4*,*7*,*8*), even when costs “are estimated liberally and the benefits presented conservatively” (*9*). Under these circumstances, infection control should be pursued, since more stands to be gained than lost (*5*). We attempt to make explicit the concepts on which these arguments rely and, in particular, concentrate on providing a framework for answering two questions: how much in total should we invest in prevention for any given infection-control situation, and how should this investment be allocated among competing infection-control strategies? Our aim is to make the economics of prevention explicit while using a minimum of technical language, algebra, and economics jargon.

## Concepts and Definitions

### Definitions of Cost

For economic analysis, the cost of using a resource should be expressed by its “opportunity cost” (*10*–*14*), the value of the next best alternative use of the resource. This can differ from the definitions used by cost accountants who work in the finance departments of hospitals. If a cost accountant calculated the cost of 500 operations, he might identify the necessary expenditures for the resources used, add them, and define this as the cost. In contrast, an economist will seek the value gained from the resources had they been used for an alternative purpose; this is the opportunity cost. An illustration of how these two approaches may produce discordant results is presented in. For example, if surgeons, operating room and equipment, nursing staff, and consumables are all procured, the cost-accountant will add up these expenditures and conclude the cost to be $565,000. However, the cost-accountant is not obliged to make ongoing payments for some of these resources. After the first 10 patients are treated, the consumables might be judged inappropriate; these expenditures can then be stopped, and the money can be redirected to other consumables. Also, if nurses are employed on a nonpermanent basis but are not meeting the required standard, they can be dismissed, and their wages can be directed toward other nurses. The $215,000 for consumables and nurses are variable costs, since we retain discretion in how this money is spent as we perform the 500 surgical procedures.

Quite different are the costs for which we are committed. If the surgeons have secure employment, then the $100,000 expenditure on their salaries cannot be avoided. Due to contractual obligations, we are committed to paying for their salaries, and the money cannot be used in any other way. Likewise, if we spend $250,000 equipping the operating room, then the money is committed and cannot be used in any other way. These costs are defined as fixed; they cannot be avoided in the short term, and we have no discretion over how this money is spent. For this reason, an economist would be wary of using the expenditure of $100,000 for surgeons and $250,000 for the operating room as the opportunity cost of these resources. Instead, the economist would seek to understand their short-term economic value in an alternative use. For example, assume that demand is such that 500 other patients are waiting to be treated for the same condition, and the first 300 patients (or their insurers) will pay $2,000 each, and the next 200 patients will pay $1,500 each. The opportunity cost of all the resources used is (300 patients x $2,000) + (200 patients x $1,500) = $900,000. By deducting the value of the opportunity costs of the nurses and consumables ($215,000), the implied economic value of the fixed costs of surgeons and operating room and equipment is $685,000. Thus, an economist would disagree with the cost-accountant and argue that the cost (the opportunity cost) of the resources listed in is $900,000. Neither the financial nor economic estimates of cost are incorrect, but the difference indicates that the healthcare sector is an imperfect economic system (*15*).

For economic analysis, the value of resources that represent variable or discretionary costs, as recorded by cost-accountants, may well represent the opportunity cost of the resources. However, the values of fixed-cost resources, as recorded by cost-accountants, are of little interest to economists. Instead, they seek the value of these resources in the short term from their alternative use, the opportunity cost.

### Perspective for Economic Evaluation

Many have argued that the benefits of infection control are widespread. Treating infection represents an economic burden, and so preventing infection potentially saves the hospital these costs (*4*,*16*–*24*); however, less is known about how preventing hospital infection offers any other benefit. One reason might be that hospital administrators, who hold the purse strings for infection control, are primarily interested in savings to their budgets and are not as focused on other benefits from preventing hospital infections that might arise for patients, informal caregivers, or other healthcare agencies (*25*). A broader perspective might include the savings from excess illness and death attributable to hospital infection. Attributing excess illness and death to hospital infection, however, is difficult, and accurately valuing these very real costs is fraught with problems. Still, when a narrow perspective is adopted and costs and benefits other than those that fall directly on the healthcare sector are excluded, economic analyses may underestimate the social benefits of infection-control programs.

### Incremental Benefits and Marginal Analysis

Incremental benefits and marginal analysis refer to adding benefit to or subtracting cost from the status quo (existing hospital expenditures and their outcomes) (*26*). If the existing budget for infection control is $100,000 and a new infection-control program costs $40,000, the total cost of infection control will increase to $140,000. The incremental cost of the infection-control program is the change in total cost from $100,000 to $140,000, or $40,000. If implementing this program avoids 50 bloodstream infections, then the incremental benefits of the new program are 50 avoided infections. Marginal analysis is similar but refers to a change of just one unit, say $1 or one infection. Most infection-control strategies would cause incremental changes, not pure marginal changes.

### Infection-Control Investment and Strategies

**Figure 1:**
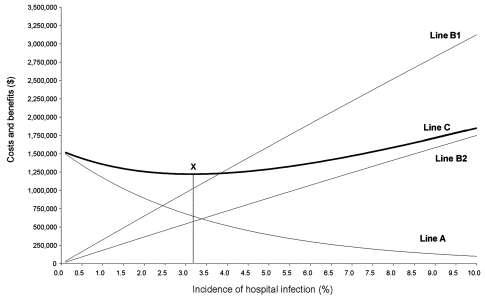
A model of investment in infection-control activities. Line A, cost and effectiveness of infection control; line B1, gross costs of infection and benefits of prevention; line B2, net costs of infection and benefits of infection control; line C, total costs; point X, incidence that minimizes total costs.

In the sections that follow, we adopt the perspective of a hospital administrator and only examine costs and savings to the hospital. We do not seek to determine a social value of the health benefits of avoiding hospital-acquired infection, so the estimate of the benefits of infection control is conservative. We also assume that all decisions are made within the short term; this is the time frame in which fixed costs cannot be changed. The model illustrated in Figure 1 uses hypothetical data to analyze the costs and benefits of prevention (*27*) and illustrate answers to both questions: 1) how much to invest for infection control and 2) which are the most appropriate infection-control programs.

### How Much To Invest for Infection Control

The horizontal axis in Figure 1 represents an incidence of wound infections in 50,000 patients undergoing hip replacement. The vertical axis represents cost and potential savings. Line A summarizes the relationship between the cost and the effectiveness of infection control strategies. To achieve the low incidence of 0.01% requires an investment of resources in infection control valued at $1.5 million. However, to reduce rates to only 5.00% requires a lesser investment of $393,661. Line B1 represents the gross costs of hospital infection, i.e., the gross savings that would result from prevention. These costs and potential savings increase with incidence. The primary cost of hospital infection is the loss of bed-days due to prolonged length of stay. Care must be taken in valuing these bed-days and other resources used for hospital infection (*28*). For economic analysis, consider what else could be done with the resources released by prevention. A hospital in which rates of infection are successfully reduced will have more bed-days available, so new patients can be admitted. The value of these new admissions to the hospital represents the gross costs of infection and, therefore, the potential gross savings from prevention. For example, if demand for hip replacement is such that patients, their insurers, or the public medical system is prepared to pay $1,250 to the hospital for each additional case treated, then the opportunity cost of wound infection is the revenue that could be earned by treating extra cases with the bed-days used by hospital infection. In Appendix 1, we give examples of how to calculate these costs for an incidence of 10.00% and 5.00%, and these data are used to plot line B1 in Figure 1.

So far we have restricted our discussion of the cost and savings from prevention to changes in the use of bed-days. We should also consider the financial expenditures made by the hospital. The financial value of resources that represent fixed costs are largely irrelevant, as they cannot be avoided in the short-term. However, fixed costs are certainly being used more productively.^2^ More relevant are the variable or discretionary costs that change in response to a decrease in the incidence of hospital infection. First, patients who previously would have stayed for 15 days with a hospital infection now stay only 10 and will incur lower variable costs.^3^ If the decrease in variable costs from reducing length of stay by 5 days is $100 per patient, then line B1 in Figure 1 is too low an estimate of the costs of infection and the potential savings from prevention. However, variable costs will also increase as a result of the increase in patient turnover. At rates of zero infection, hospitals are treating 2,500 more patients than before, and this will cause an increase in variable costs. For example, the capacity to perform the surgery will have to be increased, requiring more surgeons, anesthetists, operating room nurses, and prostheses and other consumables. If the increase in variable cost is evaluated at $750 per new admission, then this must be offset against the $100 per patient reduction in variable costs and the $1,250 increase in revenue per case. The result is the net costs of infection and net savings from prevention. In Appendix 2 we illustrate how to calculate these costs for an incidence of 10.00% and 5.00%. This suggests the gross costs of infection, so the gross savings from prevention marked by line B1 is incorrect. We indicate the correct values by line B2.

Line C in Figure 1 is the cost to the healthcare system and is the sum of lines A and B2 for every incidence rate of hospital infection. For example, at an incidence of 9.00%, the net cost of infection is $1,582,536 (Line B2), and the cost of prevention programs is $132,088 (Line A). The sum of these at an incidence of 9.00% is $1,714,624 (Line C).

The incidence of infection that minimizes the costs indicated by Line C is marked with an X in Figure 1 and represents a rational objective for policy makers. To explore this point further, consult Appendix 3, which includes the values used to plot lines A, B2, and C between the incidence rates of 2.9% and 3.4%. We conclude that point X is a rational policy goal because savings compensate for investments in prevention. In contrast, investments that drive infection rates lower than point X are not adequately compensated for. The last infection we should prevent will cost $17,810 in terms of infection-control activities but will release resources worth $17,810.

The aggregate investment that achieves the rate indicated by point X is therefore the correct budget constraint for infection control. At point X, there is no net gain or loss, which signals the best achievable, or equilibrium, outcome.

### Determining Appropriate Infection-Control Programs

There are many different ways of preventing hospital infections and therefore many different ways of moving toward point X. Choices have to be made among the numerous competing infection-control programs available. To help make these choices, we apply the technique of incremental cost-effectiveness analysis (*29*), where the costs of the interventions are represented in monetary terms, and the benefits are measured in natural units common to all interventions under consideration. For this example, the benefits of the intervention are the number of cases of infection avoided. To find the best intervention, we want to choose the infection-control activities that minimize the cost per infection avoided while remaining within the budget constraint identified previously.

**Figure 2:**
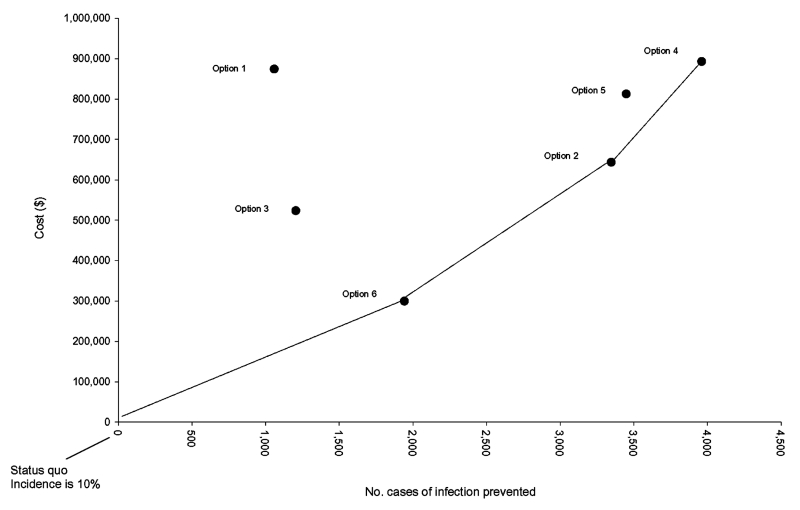
A model of cost, effectiveness, and benefits of six competing infection-control strategies.

A useful first step is to identify a patient group and an infection to prevent. Keeping with the example of infection in hip replacement, the next step is to identify all reasonable strategies that might prevent this type of infection. In our example, we propose six strategies and assume that all available prevention strategies are represented by these six options. The cost, effectiveness, and benefits of each are illustrated in the Table, and these data are plotted in Figure 2. Options 1 to 6 compete with each other, and only the most appropriate will be used.

**Table:** Cost, effectiveness, and benefits of six competing infection-control strategies

Option	Incremental cost of prevention	Incremental benefit^a^	Effect^b^	
Option 6	$299,611	1,942	4.00%	
Option 3	$523,487	1,205	2.50%	
Option 2	$643,487	3,346	6.80%	
Option 5	$812,457	3,448	7.10%	
Option 1	$874,512	1,059	2.20%	
Option 4	$892,931	3,960	8.00%	

^a^Cases prevented. ^b^Reduction in incidence.

The status quo is an incidence of 10.00% for a population of 50,000 patients who receive a new hip in a given period. Option 6 is clearly preferable to options 1 to 5 because the cost of preventing one infection by this mode is only $154, calculated by dividing the cost of option 6 by the benefit of option 6, both relative to the status quo. The cost of preventing one infection by this mode is known as the incremental cost-effectiveness ratio (ICER). See Appendix 4 to clarify how to calculate ICERs. In our example, the hospital should first invest $299,611, moving from the origin to option 6.

Now, all other options (except option 6) are still available, and any further decisions must be evaluated with respect to option 6, the new status quo. Both option 1 and option 3 are less effective and more costly than the status quo and so are excluded. Option 2 beats options 4 and 5; although all prevent further infections, option 2 does so at the lowest cost. The hospital may wish to invest a further $343,876, moving from option 6 to option 2.

The status quo is now option 2, and only options 4 and 5 remain, with the final move being to option 4. The question of which are the most effective infection-control programs has been answered. A policy represented by a line that joins the origin to the points marked option 6, option 2, and option 4 illustrates the most appropriate, most cost-effective infection-control strategy.

**Figure 3:**
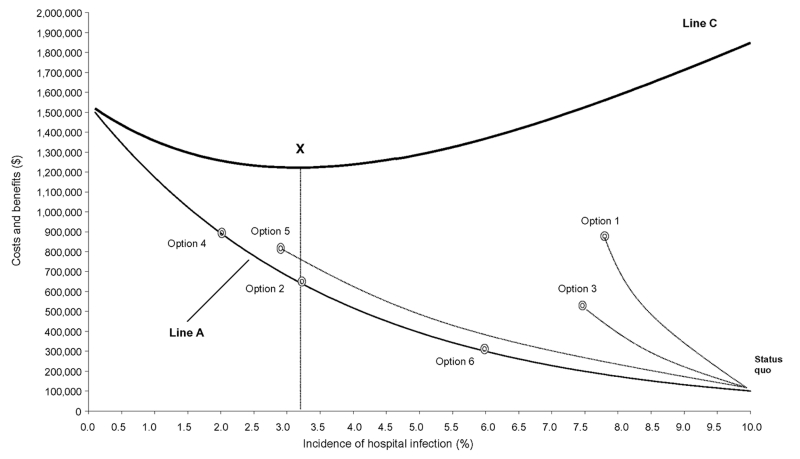
Six competing infection-control strategies imposed on the model of investment in infection-control activities.

In this example, we have pursued the most cost-effective pathway without regard to the budget constraint that minimizes costs to the healthcare system. Consider the information included in Figure 3. This is a version of Figure 1 that includes the incremental costs and benefits of the six competing strategies described above. The status quo, at an incidence of 10%, and the moves to options 6, 2, and 4 that define the cost-effective pathway are marked. The figure shows that the hospital should not invest beyond the point defined by option 2. While a further move to option 4 is the lowest cost alternative for preventing further cases of infection, option 4 exceeds budget constraints and increases costs to the healthcare system (line C).

## Discussion

Many have considered the economics of preventing hospital-acquired infection. We argue, with the exception of one study (*30*), the complexity of the economic issues has been neglected. In this article we attempt to make the economics explicit. We demonstrated how the concept of opportunity cost might be used to value the costs of hospital infection and therefore the savings from infection control programs. We argue that existing literature uses financial costs to represent the cost of infection, and this method may lead to erroneous conclusions. Financial costs are a monetized estimate value of health-services cost (*31*) and do not satisfy the definition of opportunity cost. We offer an explicit treatment of how variable costs change in response to infection control and highlight the difference between the gross and net costs of hospital infection. We also suggest that, as the perspective for analysis broadens, the costs of infection and the potential benefits of infection control increase. This will affect the position of point X in our example and, therefore, affect infection control policy. Finally, we identify a budget constraint for infection control that identifies the point where the costs of prevention are compensated by simultaneous cost-savings and illustrate how incremental cost-effectiveness analysis might be used to identify the most efficient choices for infection control.

To build the model we propose requires data to plot lines B2 and A; obtaining these data will allow line C to be estimated and point X to be identified for any given hospital infection scenario. Plotting line B2 requires data on the incidence of hospital infection and the resulting opportunity costs. Although a complicated task, progress is being made with the specification of models (*32*,*33*), and establishing the true effect of hospital infection on length of stay and cost is now a more rigorous process. Deriving values of alternative uses of these bed-days represents further challenges. Due to the absence of a reliable market mechanism for health care, finding an accurate valuation for a marginal admission to a hospital is difficult (*15*), as is finding the opportunity cost of bed-days. Further research in this area is required. Plotting line A requires that the cost and effectiveness of competing infection control strategies be understood. Although the number of economic evaluations that include an assessment of costs and benefits of infection-control strategies are limited (*34*), a broad and diverse literature exists on the effectiveness of many infection-control interventions. The quality of the evidence is likely to be variable, encompassing a range between correctly designed, randomized, controlled trials and subjective, expert opinion. If the findings could be synthesized in a rigorous manner and summary estimates of the likely effectiveness derived, the costs of these strategies could be estimated separately and the data required to plot line A procured. With data to plot lines A, B2, and C, point X can be identified. Achieving this for the numerous patient groups and sites of hospital infection will be a major task, but the conceptual framework, expertise, and data are available for an explicit treatment of the economics of preventing hospital infection.

## Supplementary Material

Appendix 1 Calculating the gross costs of hospital-acquired infection

Appendix 2 Calculating the net costs of hospital-acquired infection

Appendix 3 The values for lines A, B2, and C between incidence rates of 2.9% and 3.4

Appendix 4 Calculating Incremental Cost-Effectiveness Ratios (ICERs)

## Footnotes

1 Care should be taken in interpreting this estimate, as it was derived from data gathered in the mid-1970s for the Study on the Efficacy of Nosocomial Infection Control (SENIC).

2 At rates of 10% the fixed costs of the organization were managing to treat 50,000 patients, but at zero rates of infection they are treating 52,500 patients, representing an improvement in efficiency. See Appendix 1.

3 Reductions would be in expenditures on antimicrobials to treat the infection and on the equipment used to deliver therapy. Expenditures on resources used for wound care such as dressings, irrigations, and other consumables would be reduced. Also, workload of the nursing staff may be reduced, so expenditures on agency nurses would be less.
