# Supplementary material for: Economics and Preventing Hospital-acquired Infection
Source: Emerg Infect Dis. 2004 Apr;10(4):561–6. doi: 10.3201/eid1004.020754 (PMC3086182; doi:10.3201/eid1004.020754)
Supplement: Appendix 1 — Calculating the gross costs of hospital-acquired infection [file 02-0754-app1-s1.pdf]

Appendix 1. Calculating the gross costs of hospital-acquired infection

Assumptions

Available bed-days = 525,000

Length of stay for patients without hospital-acquired infection (HAI) = 10 days

Length of stay for patients with HAI = 15 days

Revenue earned per patient treated = $1,250

Calculations

(1) Incidence of wound infection	10%	5%	0%
(2) Total admissions[a]	50,000	51,220	52,500
(3) Number of patients that acquire HAI[b]	5,000	2,561	0
(4) Number of patients that do not acquire HAI[c]	45,000	48,659	52,500
(5) Bed-days used by those that do not acquire HAI[d]	450,000	486,590	525,000
(6) Bed-days used by those that acquire HAI[e]	75,000	38,415	0
(7) Revenue earned from all admissions[f]	$62,500,000	$64,025,000	$65,625,000
(8) Gross cost (loss of revenue due to the incidence of HAI)[g]	$3,125,000	$1,600,000	$0

[a]The number of admissions that can be treated with the 525,000 bed-days available at that incidence rate. We calculate this figure by dividing 525,000 (available bed-days) by the rate of infection times 15 days plus the rate of noninfection times 10 days. For example, at 10% incidence, 525,000 / ([10% x 15] + [90% x 10]) = 525,000 / (1.5 + 9) = 50,000.

[b]Calculated by (1) x (2).

[c]Calculated by (2) – (3).

[d]Calculated by (4) x 10 days.

[e]Calculated by (3) x 15 days.

[f]Calculated by (2) x $1,250.

[g]Calculated by ([7] at 0%) – ([7] at 10%) and ([7] at 0%) – ([7] at 5%); these data are used to plot Line B1 in Figure 1.
