# Supplementary material for: Economics and Preventing Hospital-acquired Infection
Source: Emerg Infect Dis. 2004 Apr;10(4):561–6. doi: 10.3201/eid1004.020754 (PMC3086182; doi:10.3201/eid1004.020754)
Supplement: Appendix 2 — Calculating the net costs of hospital-acquired infection [file 02-0754-app2-s2.pdf]

Appendix 2. Calculating the net costs of hospital-acquired infection

Assumptions

The change in the variable costs attributable to a case of hospital-acquired infection (HAI) is $100.

The change in variable costs attributable to a new admission is $750.

Calculations

Incidence of wound infection	10%	5%	0%
(1) Total admissions achieved[a]	50,000	51,220	52,500
(2) Extra cases that could be treated if incidence was 0%[b]	2,500	1,280	0
(3) Number that acquire HAI[a]	5,000	2,561	0
(4) Lost revenue (gross cost of HAI)[a]	$3,125,000	$1,600,000	$0
(5) Variable costs if extra cases were treated[c]	$1,875,000	$960,000	$0
(6) Variable costs for each case of infection[d]	$500,000	$256,100	$0
(7) Net cost of HAI[e]	$1,750,000	$896,100	$0

[a]See Appendix 1 for details of how this figure is derived.

[b]Calculated by ([1] at 0%) – ([1] at 10%) and ([1] at 0%) – ([1] at 5%).

[c]Calculated by (2) x $750.

[d]Calculated by (3) x $100.

[e]Calculated by (4) + (6) – (5); these data are used to plot Line B2 in Figure 1.
