# Supplementary material for: Economics and Preventing Hospital-acquired Infection
Source: Emerg Infect Dis. 2004 Apr;10(4):561–6. doi: 10.3201/eid1004.020754 (PMC3086182; doi:10.3201/eid1004.020754)
Supplement: Appendix 3 — The values for lines A, B2, and C between incidence rates of 2.9% and 3.4 [file 02-0754-app3-s3.pdf]

Appendix 3. The values for lines A, B2, and C between incidence rates of 2.9% and 3.4%

Notes

1. Reducing incidence from 3.3% to 3.2% causes the net cost of hospital infection (line B2 on Figure 1) to fall from $596,532 to $578,740, an incremental savings of $17,792.
2. The cause of this reduction in incidence from 3.3% to 3.2% is an incremental investment in prevention (line A on Figure 1). The costs of prevention rise from $626,157 to $643,487, an incremental cost of $17,330.
3. Costs have increased by $17,330 but have been offset by a saving of $17,792. Total costs (line C on Figure 1) have fallen from $1,222,689 to $1,222,227, a net saving of $462.
4. Economists would support the practices that lead to the reduction in rates from 3.3% to 3.2% as savings exceed costs by $462.

Notes

1. Reducing incidence beyond the optimum, from 3.1% to 3.0%, also reduces the net costs of hospital infection (Line B2 on Figure 1) from $560,931 to $543,103, an incremental saving of $17,827.
2. The cost of achieving this reduction is the change in the costs of prevention (Line A on Figure 1) from $661,297 to $679,599, an incremental cost of $18,302.
3. In this case, in which rates of hospital infection are lower than the optimum, as defined by point X, the costs of the reduction are not completely offset by the benefits. Total cost (Line C on Figure 1) rises from $1,222,227 to $1,222,703, an increase of $476.
4. Although infection rates are further reduced, economists would not support the practices that lead to this reduction in incidence from 3.1% to 3.0%. More has been lost than has been gained with costs exceeding savings by $476.

Data

Incidence	Net cost of infection and potential cost saving (line B2)	Cost of prevention (line A)	Total cost (line C)	Incremental cost saving	Incremental cost	Change in total cost
2.90%	$525,259	$698,408	$1,223,667			
				$17,845	$18,809	$964
3.00%	$543,103	$679,599	$1,222,703			
				$17,827	$18,302	$476
**3.10% (point X)**	**$560,931**	**$661,297**	**$1,222,227**			
				**$17,810**	**$17,810**	**$0**
3.20%	$578,740	$643,487	$1,222,227			
				$17,792	$17,330	–$462
3.30%	$596,532	$626,157	$1,222,689			
				$17,775	$16,863	–$912
3.40%	$614,307	$609,294	$1,223,601			
