# Supplementary material for: Economics and Preventing Hospital-acquired Infection
Source: Emerg Infect Dis. 2004 Apr;10(4):561–6. doi: 10.3201/eid1004.020754 (PMC3086182; doi:10.3201/eid1004.020754)
Supplement: Appendix 4 — Calculating Incremental Cost-Effectiveness Ratios (ICERs) [file 02-0754-app4-s4.pdf]

Appendix 4. Calculating Incremental Cost-Effectiveness Ratios (ICERs)

Compared to Status Quo

ICER for option 6 as compared to status quo = $154[a]

$$\frac{\text{IC}^{b}\text{ of option 6 (\$299,611) – IC of the status quo (\$0)}}{\text{IB}^{b}\text{ of option 6 (1,942 patients) –IB of the status quo (0 patients)}}$$

ICER for option 3 as compared to status quo = $434

$$\frac{\text{IC of option 3 (\$523,487) – IC of the status quo (\$0)}}{\text{IB of option 3 (1,205 patients) – IB of the status quo (0 patients)}}$$

ICER for option 2 as compared to status quo = $192

$$\frac{\text{IC of option 2 (\$643,487) – IC of the status quo (\$0)}}{\text{IB of option 2 (3,346 patients) – IB of the status quo (0 patients)}}$$

ICER for option 5 as compared to status quo = $236

$$\frac{\text{IC of option 5 (\$812,457) – IC of the status quo (\$0)}}{\text{IB of option 5 (3,348 patients) – IB of the status quo (0 patients)}}$$

ICER for option 1 as compared to status quo = $826

$$\frac{\text{IC of option 1 (\$874,512) – IC of the status quo (\$0)}}{\text{IB of option 1 (1,059 patients) – IB of the status quo (0 patients)}}$$

ICER for option 4 as compared to status quo = $225

$$\frac{\text{IC of option 6 (\$892,931) – IC of the status quo (\$0)}}{\text{IB of option 6 (3,960 patients) – IB of the status quo (0 patients)}}$$

Compared to Option 6

ICER for option 3 as compared to option 6[c] = –$304

$$\frac{\text{IC of option 3 (\$523,487) – IC of option 6 (\$299,611)}}{\text{IB of option 3 (1,205 patients) – IB of option 6 (1,942 patients)}}$$

ICER for option 2 as compared to option 6[d] = $245

$$\frac{\text{IC of option 2 (\$643,487) – IC of option 6 (\$299,611)}}{\text{IB of option 2 (3,346 patients) – IB of option 6 (1,942 patients)}}$$

ICER for option 5 as compared to option 6 = $340

$$\frac{\text{IC of option 5 (\$812,457) – IC of option 6 (\$299,611)}}{\text{IB of option 5 (3,348 patients) – IB of option 6 (1,942 patients)}}$$

ICER for option 1 as compared to option 6[c] = –$651

$$\frac{\text{IC of option 1 (\$874,512) – IC of option 6 (\$299,611)}}{\text{IB of option 1 (1,059 patients) – IB of option 6 (1,942 patients)}}$$

ICER for option 4 as compared to option 6 = $294

$$\frac{\text{IC of option 6 (\$892,931) – IC of option 6 (\$299,611)}}{\text{IB of option 6 (3,960 patients) – IB of option 6 (1,942 patients)}}$$

Compared to Option 2

ICER for option 5 as compared to option 2 = $1,664

$$\frac{\text{IC of option 5 (\$812,457) – IC of option 2 (\$643,487)}}{\text{IB of Option 5 (3,448 patients) – IB of the Option 2 (3,346 patients)}}$$

ICER for option 4 as compared to option 6[e] = $406

$$\frac{\text{IC of option 4 (\$892,931) – IC of option 2 (\$643,487)}}{\text{IB of option 4 (3,960 patients) – IB of option 2 (3,346 patients)}}$$

[a]The most cost-effective as compared to status quo.

[b]IC, incremental cost; IB, incremental benefit.

[c]More costly and less effective than another available option.

[d]The most cost-effective as compared to option 6.

[e]The most cost-effective as compared to option 2.
